# Prevalence and determinants of burnout among South African doctors during the COVID-19 pandemic

**DOI:** 10.4102/sajpsychiatry.v30i0.2225

**Published:** 2024-04-08

**Authors:** Saajida Khan, Itumeleng Ntatamala, Roslynn Baatjies, Shahieda Adams

**Affiliations:** 1 Division of Occupational Medicine and Centre for Environmental and Occupational Health Research, School of Public Health, University of Cape Town, Cape Town, South Africa; 2 Department of Environmental and Occupational Studies, Faculty of Applied Sciences, Cape Peninsula University of Technology, Cape Town, South Africa

**Keywords:** burnout, work-related stress, medical doctors, COVID-19, public hospital, South Africa

## Abstract

**Background:**

Burnout, resulting from chronic workplace stress that has been unsuccessfully managed, has previously been documented in doctors. The coronavirus disease 2019 (COVID-19) pandemic has increased occupational challenges faced by doctors, potentiating their risk for burnout.

**Aim:**

This study aimed to determine the prevalence and determinants of burnout among medical doctors during the COVID-19 pandemic.

**Setting:**

Three public sector hospitals in Gqeberha, South Africa.

**Methods:**

A cross-sectional study of 260 voluntary participants was conducted. Participants completed self-administered electronic questionnaires. Logistic regression analysis was performed to explore the determinants of burnout.

**Results:**

The prevalence of burnout in this study was 78%. Burnout was significantly associated with being a medical intern or community-service medical officer (adjusted odd ratio [AOR] = 6.72, 1.71–26.40), being in the lowest income band (AOR = 10.78, 2.55–45.49), and using alcohol to manage work-related stress (AOR = 3.01, 1.12–8.04). Job-related factors associated with burnout were experiencing high conflict at work (AOR = 5.04, 1.92–13.20) and high role ambiguity and role conflict (AOR = 4.49, 1.98–10.18). Low support at work (AOR = 9.99, 3.66–27.23), medium job satisfaction (AOR = 5.38, 2.65–10.93) and medium support at work (AOR = 3.39, 1.71–6.73) were positively associated with burnout. Participants with medium (AOR = 0.28, 0.10–0.80) and high levels of resilience (AOR = 0.08, 0.03–0.25) were protected against burnout. Coronavirus disease 2019-related factors were not significantly associated with burnout.

**Conclusion:**

The burnout prevalence among South African medical doctors at public hospitals during the COVID-19 pandemic was high and strongly associated with job stress factors.

**Contribution:**

Given the increased prevalence of burnout among doctors and the strong associations with job stress factors, mitigation of burnout requires targeted organisational interventions.

## Introduction

Burnout has been defined as a syndrome resulting from chronic workplace stress that has been unsuccessfully managed and is characterised by feelings of exhaustion, cynicism and reduced professional efficacy.^1^ Medical doctors have been shown to be at increased risk of burnout in comparison to other professionals.^2,3^ Because of the global spread of coronavirus disease 2019 (COVID-19), doctors had to contend with high workloads, fears of infection, increased levels of uncertainty resulting from redeployment, inadequate supply of personal protective equipment (PPE) and insufficient support, potentiating their risk of burnout.^4,5,6,7,8,9^

While burnout in doctors has been extensively studied, an existing problem identified in the literature has been that the prevalence of burnout globally and locally, both prior to and during the pandemic has remained highly variable.^10,11^ This variability may be because of the heterogeneity of methods used to assess burnout, the differing study populations involving various categories of healthcare workers (HCWs), and diverse contexts in which the participants live and work.^12^

Burnout occurs because of the interaction between occupational and individual factors.^2,13^ Individual factors associated with burnout include sociodemographic characteristics (age, gender, marital status, level of education and years employed in a post), personality type, and level of social support which are not easily modifiable through workplace interventions.^14,15,16,17^ Doctors working in frontline specialities, such as emergency medicine, internal medicine and family medicine have been found to be at greatest risk of burnout.^18,19^ Although burnout is a state related to stress, specific biomarkers could not conclusively be associated with burnout.^20^ Occupational factors contributing to burnout in doctors have been described as job demands (which include increased workload, shift work, moral injury, administrative burden and time pressure) and job resources (characterised by supervisory and peer support, salaries, career development, organisational culture, job control and increased decision latitude).^14^ The hierarchical nature of the medical profession with low levels of organisational support, appears to be related to burnout.^14,21^ Doctors disclosing physical, mental health or substance abuse challenges are faced with stigma, discrimination as well as fear of professional consequences^22^ which perpetuates poor help-seeking behaviour.^22,23,24,25^ Despite studies suggesting that both individual and occupational factors are associated with burnout, an identified problem is that healthcare institutions continue to presume that the cause of burnout lay with the worker and the solution has only focussed on fixing the individual rather than modifying the job.^26,27,28^ Consequently, there has been a call for institutions to mitigate burnout with both individual-focussed and organisational interventions.^5,8,29^

The impact of burnout on doctors has significant consequences for the individual doctor, patients and healthcare organisations.^5^ Personal consequences include increased occupational injuries, gastrointestinal disorders, sleep disturbances, substance abuse and negative impacts on mental wellbeing, resulting in depression, anxiety and increased risk of suicidal ideation.^9^ Burnout in doctors has substantial effects on patients and has been associated with increased risks of medication and diagnostic errors, poor physician-patient rapport and poor patient outcomes.^16,19^ Burnout also has negative consequences for both organisations and health systems, and is related to low productivity, increased absenteeism and presenteeism, increased malpractice litigation, and increased staff turnover in healthcare institutions.^16,19,26^

The Eastern Cape province is the second largest and one of the most rural of South Africa’s nine provinces. Because of healthcare service delivery demands, lack of resources and provincial inefficiencies, doctors in the public health sector in this province are faced daily with workplace challenges.^30,31,32^ By negatively impacting the capacity and resources of healthcare system, the COVID-19 pandemic has exacerbated the occupational stressors faced by these doctors. While studies have described the prevalence of burnout among social workers and teachers in this province,^33,34^ there exists a gap in knowledge regarding the prevalence and determinants of burnout in doctors in the Eastern Cape and interventions which would successfully reduce burnout in doctors. This cross-sectional study aimed to determine the prevalence and determinants of burnout in doctors at public hospitals in Gqeberha, a town in the Eastern Cape province, during the COVID-19 pandemic. It further assessed the resilience level in these doctors during the pandemic, with the objective of proposing preventative interventions at an organisational level that would successfully reduce burnout in doctors.

## Research methods and design

### Study design, population and sampling

A cross-sectional study was performed at three hospitals in Gqeberha which employed a total of 430 doctors. The doctors consisted of medical interns, community service medical officers, medical officers, registrars or residents, specialist consultants and clinical managers. Inclusion criteria included all medical doctors who were working at the three largest referral hospitals in Gqeberha, during April 2022 – May 2022 (the fifth wave of COVID-19 in South Africa). Doctors who were not employed at a participating hospital or did not work during the COVID-19 pandemic or study period did not form part of the sample.

There were no average estimates for burnout in doctors in sub-Saharan Africa. We used conservative estimates from the South African studies, and those that yielded prevalences of > 50% were not used in the sample size calculation. A minimum sample size of 255 was chosen for this study, to yield an anticipated estimated prevalence of burnout of 25% (α = 0.05 and power 80%).

Using REDCap electronic data capture tools hosted at the University of Cape Town,^35^ an invitation to engage in this study was emailed to the doctors, with an attached link to the questionnaire during April 2022 and May 2022. Electronic informed consent was obtained from all participants before they could access the questionnaire. Participant’s involvement in the study was voluntary, and confidentiality was guaranteed. The majority of data were captured via the online tools. However, a WhatsApp version was also provided for those participants who could not access the electronic tool. For a few participants, hardcopy questionnaires were provided, and these were placed in sealed envelopes and collected by the researcher.

A list of mental health resources was included in the email should participants feel the need to consult a healthcare provider following completion of the survey. The Institutional Review Board at a Western Cape University approved the research.

### Measurements

#### Questionnaire

A questionnaire was administered that recorded demographic information, including age, gender, marital status, number of children, caregiver status and personal risk behaviour variables (smoking, alcohol use and use of illicit or prescription drugs). Participants also provided details on job-related variables such as: occupation, occupational rank, medical or surgical specialty, service years, salary, overtime worked, overtime hours worked per month, length of overtime call, overtime site and change in job on past 2 years.

#### Assessment of burnout

The Oldenburg Burnout Inventory (OLBI) is a 16-item validated survey, based on the job demands-resources model.^36^ The OLBI covers two dimensions (exhaustion and disengagement) processed separately as a continuous variable.^37^ The OLBI consists of multiple questions for each dimension. It allows for responses in a four-point Likert scale that ranges from strongly agree (1) to strongly disagree (4). The OLBI includes both positively and negatively framed items, which improves psychometric balancing of the OLBI and reduces the likelihood of answering bias.^36^ The 16 items are divided between two subscales; the items are also summed to form two sub-totals. For negatively framed items, the scale is reversed, with ‘strongly agree’ answers scoring 4 and ‘strongly disagree’ answers scoring 1. The total score for the OLBI is the sum of the scores for the exhaustion and disengagement subscales. A higher score indicated a higher level of burnout. The mean score for the OLBI domains were obtained by totalling the scores for each item in the domain and then dividing the total scores of the items in the domain by eight. For the burnout group, participants with mean scores ≥ 2.25 on the exhaustion domain were regarded as having high exhaustion, while those participants who scored < 2.25 were considered to have low exhaustion. For the disengagement group, mean scores ≥ 2.1 were defined as high disengagement and mean scores < 2.1 were defined as low disengagement. Professional efficacy or accomplishment is not included in the OLBI as it is considered to be the weakest burnout dimension.^36^

#### Assessment of workplace stress

Work stress was measured by using the Generic Job Stress Questionnaire (GJSQ), a validated tool, which was developed by the National Institute for Occupational Safety and Health (NIOSH).^38,39^ The NIOSH permits that subscales of GJSQ be used independently to determine work stress. This study focussed on five subscales (workload and responsibility, conflict at work, role ambiguity and role conflict, job satisfaction, and support at work) to evaluate occupational stress. In the GJSQ, some items, like job satisfaction and support at work, are positively oriented with high scores indicating lower stress levels. In contrast, conflict at work and job ambiguity are negatively oriented items and high scores represent higher stress levels.^38,39^

#### Assessment of resilience

To measure resilience, this study used the Connor-Davidson Resilience Scale (CD-RISC) which has 10-items and uses a 5-point Likert scale that ranges from not true at all (0) to true nearly all of the time (4).^40^ Greater resilience was implied by higher scores. Total scores range from a minimum of 0 to a maximum of 40 and scores < 35 were considered low resilience, scores from 35 to 40 were considered medium resilience, and scores > 40 were considered high resilience. The CD-RISC has previously been reported as a reliable and efficient measure of resilience.^40^

#### Coronavirus disease 2019 related variables

Coronavirus disease 2019-related questions included details on previous COVID-19 diagnosis, transmission of COVID-19 to family and friends. Workplace interventions regarding the provision of PPE and infection prevention and control (IPC), redeployment to other workstations, and the cancelling of annual leave were also explored.

#### Interventions to reduce stress at work

To address interventions to reduce stress at work, participants chose from a list of 13 interventions, derived from the literature, relating to job demands and job resources, which would assist with reducing stress at work.

### Statistical analysis

All the data collected were analysed using Stata 15.0 statistical computer software (StataCorp, College Station, Texas, USA). The associations of interest were between independent variables (sociodemographic, occupational and environmental predictors, general and mental health status, aspects of COVID-19 infection, and workplace interventions) and burnout as the outcome variable. Logistic regression analysis was conducted to explore the association between the key outcome of burnout and environmental and work-related variables. Multivariable logistic regression models were adjusted for age and gender since these variables appeared to be the most consistent significant potential confounders identified in the univariable models. The relationship between burnout and continuous variables (resilience and job stress factors) was further explored by categorising the variable into tertiles (high, medium and low) while four categories were created for age and burnout. The burnout category represented the main outcome of interest.

### Ethical considerations

The study was conducted in accordance with the Declaration of Helsinki and approved by the Human Research Ethics Committee of the University of Cape Town (HREC 616/2021) prior to the study being conducted. All participants in the study provided written informed consent.

## Results

### Sociodemographic characteristics of the study participants

Out of 430 eligible candidates, a total of 296 doctors participated representing a response rate of 68%. However, 36 questionnaires were incomplete and not used in the analysis. Characteristics of those who did not complete the questionnaire were not significantly different to those who completed it. Therefore, 260 questionnaires were analysed for the study (Table 1).

**TABLE 1:** Sociodemographic characteristics of the study participants (*N* = 260).

Participant characteristics	*n*	%
**Gender**		
Man	109	41.92
Woman	150	57.69
Gender non-conforming	1	0.38
**Age (in years)**		
20–29	111	42.69
30–39	73	28.08
40–49	50	19.23
> 50	26	10.00
**Relationship status**		
Married	117	45.00
Single	101	38.85
In a committed relationship	42	16.15
**Children living at home**		
Yes	99	38.08
No	161	61.92
**Primary caregiver of children**		
Yes	79	79.80
No	20	20.20
**Primary caregiver of elderly or disabled family members**		
Yes	26	10.00
No	234	90.00
**Job title**		
Medical Intern	84	32.31
Community Service Medical Officer	2	0.77
Medical Officer	76	29.23
Registrar	39	15.00
Medical Specialist or Consultant	54	20.77
Clinical Manager	5	1.92
**Speciality**		
Internal Medicine†	54	20.76
Surgical‡	53	20.38
Anaesthetics	22	8.46
Family Medicine	35	13.46
Emergency Medicine	22	8.46
Obstetrics & Gynaecology	31	11.92
Paediatrics	41	15.76
Administrative	2	0.76
**Employment**		
Full-time	250	96.15
Part-time	10	3.80
**Monthly salary ($)**		
1646.00–2744.00	89	34.23
2744.00–5488.00	145	55.77
> 5488.00	26	10.00

† , Internal Medicine speciality comprised of Internal Medicine, Neurology, Psychiatry, Intensive Care Unit (ICU), Radiology, Oncology, Dermatology and Haematology.

‡ , Surgical speciality comprised of General surgery, Urology, Orthopaedic surgery, Ophthalmology, ENT and Maxillofacial surgery.

Table 1 outlines the demographic characteristics of the study population. Participants were predominantly female (58%) and young, with 43% of the participants in the age group 20–29 years. Majority of the respondents were English-speaking (66%) and less than half were married (45%). Almost 40% of subjects had children living at home and of these, 80% assumed the role of primary caregiver for the children. A few respondents (10%) were primary caregivers for elderly or disabled family members.

Junior doctors, such as medical interns (32%) and medical officers (29%), formed the dominant occupational group while medical specialists or consultants comprised 21%. The median years of service were 2 years (interquartile range [IQR] = 1–5) and 96% of the participants reported working full-time. Most worked overtime (96%) with 45% reported working between 60 and 80 h overtime per month with 67% performing their overtime on-site (Table 1).

### Mental health factors and substance use by participants

Mental health disorders were highly prevalent with 25% of the participants reporting a diagnosis of a mental health condition while 18% reported being on treatment for a mental health condition currently. The most commonly diagnosed mental health disorders in these participants were depression (41%), depression with anxiety (18%), and generalised anxiety disorder (17%). Only 8% had a prior diagnosis of burnout. To assist with the management of work-related stress (WRS), participants used different substances, which included, cigarette smoking (17%), drinking alcohol (21%), illicit drug use (5%), and the use of non-prescription medication (7%). Using the OLBI, the prevalence of burnout (as defined by a cut-off score of > 2.1 for disengagement and > 2.25 for exhaustion) in the participants was 78% (Table 2).

**TABLE 2:** Mental health factors and substance use by participants (*N* = 260).

Participant characteristics	*n*	%
**Smoking history**		
Never	213	81.92
Previous smoker	24	9.23
Current	23	8.85
**Alcohol history**		
Never	54	20.77
Previous alcohol use	40	15.38
Current	166	63.85
**Illicit drug use**		
Never	239	91.92
Previous illicit drug use	14	5.38
Current	7	2.69
**Substance use to manage work related stress (WRS)**		
Feel need to smoke to manage WRS	45	17.31
Non-prescription drug use to manage WRS	17	6.59
Feel need to drink alcohol to manage WRS	53	20.62
Feel need to use illicit drugs to manage WRS	13	5.10
**Mental health**		
Ever diagnosed with a mental health condition	66	25.48
Diagnosed with mental health condition in last 12 months	27	10.38
Currently on treatment for mental health condition	46	17.69
**Prevalence of burnout†**		
Burnout	203	78.08
Exhausted	23	8.85
Disengaged	11	4.23
Non-burnout	23	8.85

*Source:* Nwosu AD, Ossai EN, Mba UC, et al. Physician burnout in Nigeria: A multicentre generalized, cross-sectional study. BMC Health Serv Res. 2020;20(1):1–9. https://doi.org/10.1186/s12913-020-05710-8

Note: High exhaustion and high disengagement = Burnout group; High exhaustion and low disengagement = Exhausted group; Low exhaustion and high disengagement = Disengaged group; Low exhaustion and low disengagement = Non-burnout group.

† , The mean score for the OLBI domains were obtained by totalling the scores for each item in the domain and then dividing the total scores of the items in the domain by eight. For the burnout group, participants with mean scores ≥ 2.25 on the exhaustion domain were regarded as having high exhaustion, while those participants who scored < 2.25 were considered to have low exhaustion. For the disengagement group, mean scores ≥ 2.1 were defined as high disengagement and those scores < 2.1 were defined as low disengagement.

### Aspects of coronavirus disease 2019 and workplace interventions

The evaluation of COVID-19 impacts revealed that over half of the participants had been infected with COVID-19 (56%). A small number of the participants (6%) were still experiencing symptoms, with almost half (49%) reporting a full recovery. Some participants reported transmitting COVID-19 to family members (18%) and 38% had to care for family members who had contracted COVID-19. Workplace interventions to mitigate COVID-19 risk such as the adequate supply of PPE (49%) and adequate implementation of IPC measures and policies in their workplaces (45%) were also reported, with majority (93%) also having received vaccination against COVID-19 (Table 3).

**TABLE 3:** Prevalence and management of coronavirus disease 2019 infections.

Aspects of COVID-19	*N*	%
**Infection and transmission**		
Infected with COVID-19	146	56.15
Fully recovered from COVID-19 infection	128	49.23
Still experiencing symptoms	16	6.15
Transmitted COVID-19 infections to family members	47	18.36
Had to care for family members who contracted COVID-19	100	38.46
**Workplace interventions**		
Given an adequate supply of PPE during COVID-19 pandemic	127	48.85
Adequate implementation of IPC measures and policies	117	45.00
Redeployed to another department during pandemic	60	23.08
Had to change annual leave because of COVID-19	147	56.54
Received vaccination against COVID-19	244	93.85

COVID-19, coronavirus disease 2019; PPE, personal protective equipment; IPC, infection prevention and control.

### Interventions proposed by participants to reduce stress at work

**FIGURE 1:**
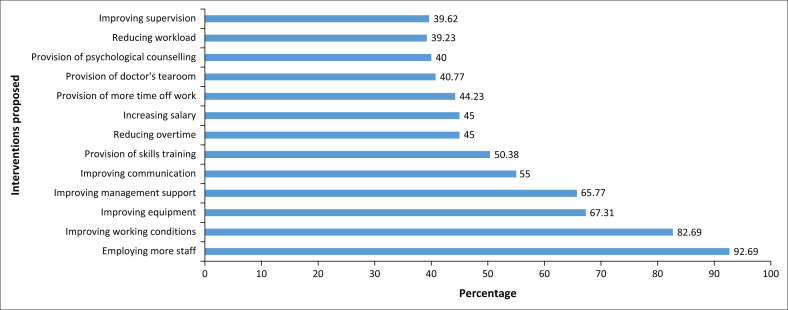
Interventions proposed by participants to reduce stress at work.

Participants supported organisational measures to mitigate workplace-related stress such as employing more staff (93%), improving working conditions (83%), improving equipment supplies (67%), and improving management support (66%). Other measures such as improving communication and the need for skill training were also supported. The least supported interventions (39%) were reducing workload or improving supervision (Figure 1).

### Job stress factors and resilience

Half of the participants (50%) experienced medium levels of workload and responsibility. Over a quarter of the participants (29%) experienced high levels of conflict at work and a third (34%) experienced high levels of role ambiguity and role conflict. Low job satisfaction (32%) and low levels of support (24%) were also reported. Based on the CD-RISC score, 25% of the participants had high levels of resilience while 46% had medium resilience and the remaining quarter had low levels of resilience (Table 4).

**TABLE 4:** Frequency table for National Institute for Occupational Safety and Health Generic Job Stress Questionnaire subscales and Connor-Davidson Resilience Scale (according to tertiles).

Risk factors	*n*	%
**NIOSH GJSQ**		
**Workload and responsibility**		
Low (14)	81	31.15
Medium (14–16)	129	49.62
High (> 16)	50	19.23
**Conflict at work** †		
Low (< 9)	64	24.62
Medium (9–13)	121	46.54
High (> 13)	75	28.85
**Role ambiguity and role conflict**		
Low (< 13)	84	32.31
Medium (13–16.26)	88	33.85
High (> 16.26)	88	33.85
**Job satisfaction ‡**		
Low (< 7)	83	31.92
Medium (7–9)	114	43.85
High (> 9)	63	24.23
**Support at work ‡**		
Low (< 7)	62	23.85
Medium (7–10)	123	47.31
High (> 10)	75	28.85
**Resilience (CD-RISC score)**		
Low (< 35)	75	28.85
Medium (35–40)	120	46.15
High (> 40)	65	25.00

NIOSH, National Institute for Occupational Safety and Health; GJSQ, Generic Job Stress Questionnaire; CD-RISC, Connor-Davidson Resilience Scale.

† , Conflict at work (Questions on Intergroup conflict and Intragroup conflict combined).

‡ , Job satisfaction and Support at work reverse coded to reflect increased risk with increased scoring category.

### Logistic regression analyses

#### Unadjusted logistic regression analysis

Unadjusted logistic regression analysis was performed for this study (Table 5) followed by multivariable analysis with each model adjusted for age and gender.

**TABLE 5:** Unadjusted and adjusted regression analysis for the correlates of burnout: Demographic and general health characteristics of the participants, resilience, job stress and aspects of coronavirus disease 2019 and workplace interventions (*N* = 260).

Correlates	Unadjusted analysis			Adjusted multivariable analysis†		
	OR	95% CI	*P*	OR	95% CI	*P*
**Job title or category**						
Medical Specialist or Consultant	1.00	-	-	-	-	-
Medical Officer	1.07	0.48–2.41	0.85	1.82	0.63–5.22	0.26
Registrar	0.62	0.25–1.52	0.29	0.47	0.14–1.54	0.21
Medical Intern‡	1.74	0.75–4.05	0.19	**6.72**	**1.71–26.40**	**0.006**
**Department and/or speciality**						
Anaesthetics	1.00	-	-	-	-	-
Paediatrics	0.68	0.18–2.51	0.57	0.72	0.19–2.67	0.62
Emergency and Family Medicine	1.58	0.41–6.07	0.50	2.10	0.51–8.55	0.30
Surgical and Obstetrics and Gynaecology	0.55	0.17–1.81	0.33	0.61	0.18–2.04	0.43
Internal Medicine	0.77	0.22–2.73	0.69	0.80	0.22–2.88	0.74
**Overtime**						
No	1.00	-	-	-	-	-
Yes	**4.78**	**1.24–18.44**	**0.02**	4.01	0.93–17.23	0.06
**Monthly salary (ZAR)**						
> 100 000	1.00	-	-	-	-	-
50 000–100 000	1.60	0.65–3.90	0.30	2.40	0.76–7.63	0.13
30 000–50 000	**3.39**	**1.23–9.33**	**0.01**	**10.78**	**2.55–45.49**	**0.001**
**Substance use to manage work related stress (WRS)**						
Feel need to smoke to manage WRS	1.14	0.51–2.55	0.73	1.16	0.51–2.63	0.70
Feel need to use alcohol to manage WRS	**3.2**	**1.20–8.47**	**0.019**	**3.01**	**1.12–8.04**	**0.02**
Feel need to use illicit drugs to manage WRS	NC	-	-	NC	-	-
Feel need to use prescription drugs to manage WRS	4.84	0.62–37.33	0.13	5.01	0.64–39.14	0.12
**Mental health**						
Ever diagnosed with mental health diagnosis	1.08	0.55–2.14	0.81	1.10	0.54–2.23	0.77
Diagnosed with mental health diagnosis last year	2.41	0.69–8.32	0.16	3.53	0.80–15.47	0.09
On treatment for mental health diagnosis	1.18	0.53–2.63	0.67	1.28	0.55–2.96	0.56
**Resilience (CD-RISC score)**						
Low	1.00	-	-	-	-	-
Medium	**0.28**	**0.10–0.78**	**0.015**	**0.28**	**0.10–0.80**	**0.01**
High	**0.09**	**0.03–0.26**	**0.000**	**0.08**	**0.03–0.25**	**0.000**
**Job stress**						
**Workload and responsibility**						
Low	1.00	-	-	-	-	-
Medium	1.53	0.78–2.98	0.21	1.44	0.72–2.86	0.29
High	1.10	0.48–2.50	0.80	1.06	0.44–2.51	0.89
**Conflict at work**						
Low	1.00	-	-	-	-	-
Medium	1.73	0.89–3.38	0.10	1.69	0.85–3.35	0.12
High	**5.08**	**2.00–12.94**	**0.001**	**5.04**	**1.92–13.20**	**0.001**
**Role ambiguity and conflict**						
Low	1.00	-	-	-	-	-
Medium	**2.32**	**1.16–4.63**	**0.017**	**2.25**	**1.11–4.57**	**0.02**
High	**4.33**	**1.95–9.59**	**0.000**	**4.49**	**1.98–10.18**	**0.000**
**Job satisfaction**						
High	1.00	-	-	-	-	-
Medium	**4.81**	**2.45–9.43**	**0.000**	**5.38**	**2.65–10.93**	**0.000**
Low	**25.75**	**5.90–112.37**	**0.000**	**27.82**	**6.27–123.45**	**0.000**
**Support at work**						
High	1.00	-	-	-	-	-
Medium	**3.18**	**1.62–6.22**	**0.001**	**3.39**	**1.71–6.73**	**0.000**
Low	**8.87**	**3.35–23.48**	**0.000**	**9.99**	**3.66–27.23**	**0.000**
**Aspects of COVID-19**						
**Infection and transmission**						
Infected with COVID-19	1.2	066–2.16	0.54	1.11	0.60–2.04	0.72
Fully recovered from COVID-19 infection	0.44	0.09–2.05	0.30	0.96	0.52–1.74	0.89
Still experiencing symptoms	15	0.48–464.20	0.12	4.15	0.51–33.13	0.17
Transmitted COVID-19 infections to family members	2.14	0.86–5.35	0.10	2.11	0.84–5.30	0.11
Had to care for family members who contracted COVID-19	1.32	0.71–2.46	0.36	1.33	0.70–2.51	0.37
Given an adequate supply of PPE during COVID-19 pandemic	0.62	0.34–1.13	0.12	0.59	0.32–1.09	0.09
Adequate implementation of IPC measures and policies	0.73	0.41–1.33	0.31	0.78	0.43–1.42	0.42
Redeployed to another department during pandemic	0.89	0.45–1.78	0.76	0.83	0.41–1.68	0.61
Had to change annual leave because of COVID-19	0.93	0.51–1.68	0.81	0.80	0.40–1.61	0.54
Received vaccination against COVID-19	1.20	0.37–3.87	0.75	1.26	0.38–4.13	0.70

Note: Statistically significant results indicated in bold.

OR, odds ratio; CI, confidence interval; COVID-19, coronavirus disease 2019; NC, non-computable; WRS, work-related stress; CD-RISC, Connor-Davidson Resilience Scale; PPE, Personal Protective Equipment.

† , Data adjusted for age and gender.

‡ , Community service medical officers and interns were grouped together as both groups had less than 4 years’ experience.

Burnout was significantly associated with working overtime (OR = 4.78,1.24–18.44). Following adjustment, the association between burnout and working overtime was attenuated and no longer significant. However, some associations between burnout and associated factors were strengthened after adjusted analysis. These associations involved being in the lowest income band (OR = 3.39, 1.23–9.33), as well as feeling the need to use alcohol to manage WRS (OR = 3.2, 1.20–8.47).

Significant job stress predictors were reporting high conflict at work (OR = 5.08, 2.00–12.94), high role ambiguity and conflict (OR = 4.33, 1.95–9.59). Participants with medium role ambiguity and conflict (OR = 2.32, 1.16–4.63) were also more likely to have burnout. Low job satisfaction (OR = 25.75, 5.90–112.37), low support at work (OR = 8,87, 3.35–23.48), medium job satisfaction (OR = 4.81, 2.45–9.43) and medium support at work (OR = 3.18, 1.62–6.22) were also positively associated with burnout.

Medium (OR = 0.28, 0.10–0.78) and high resilience (OR = 0.09, 0.03–0.26) were found to be significantly protective against burnout. The analysis of aspects of COVID-19 infection associated with burnout did not reveal any significant associations in the unadjusted analysis.

#### Adjusted multivariable logistic regression analysis

Multivariable regression analysis was computed (Table 5), with each model adjusted for age and gender. Burnout was significantly associated with being a medical intern or community service medical officer (AOR = 6.72, 1.71–26.40). There were few associations between burnout and some factors, which strengthened after adjusted analysis. These associations involved being in the lowest income band (AOR = 10.78, 2.55–45.49) as well as feeling the need to use alcohol to manage WRS (AOR = 3.01, 1.12–8.04).

Regarding the job stress predictors, burnout was significantly associated with high conflict at work (AOR = 5.04, 1.92–13.20) and high role ambiguity and role conflict (AOR = 4.49, 1.98–10.18). Participants with medium role ambiguity and role conflict (AOR = 2.25, 1.11–4.57) were also more likely to have burnout. Low job satisfaction (AOR = 27.82, 6.27–123.45), low support at work (AOR = 9.99, 3.66–27.23), medium job satisfaction (AOR = 5.38, 2.65–10.93) and medium support at work (AOR = 3.39, 1.71–6.73) were positively associated with burnout.

With multivariable regression analysis, medium resilience (AOR = 0.28, 0.10–0.80) and high resilience (AOR = 0.08, 0.03–0.25) were found to be significantly protective against burnout. While some factors related to COVID-19 infection and workplace interventions were associated with burnout, none of these remained statistically significant following adjusted analysis.

## Discussion

This study found the prevalence of burnout among medical doctors at public hospitals in Gqeberha, South Africa, during the COVID-19 pandemic to be high at 78%. A strong association was shown between burnout and being a junior doctor and between burnout and substance use. While work-related factors such as conflict at work, role ambiguity and role conflict were positively associated with burnout, other occupational factors like support at work, job satisfaction and resilience were protective.

While the prevalence of burnout in this study is higher than global trends,^18,41^ it is consistent with the high burnout prevalence found in some local studies, conducted before the pandemic, which ranged from 59% to 84%.^3,15,40,42,43,44^ Previous studies in other low- and middle-income countries (LMICs) observed high variability in prevalence rates between 62% and 95%.^10,12^ The prevalence of burnout in this study is consistent with the 75.5% found in a cross-sectional study conducted in Nigeria, prior to the pandemic, which also used the OLBI and matching cut-off scales.^36^

This study supports evidence from previous studies^3,18,44^ that showed no significant association between burnout and sociodemographic factors such as age, gender, relationship status, and being the primary caregiver of children or elderly or disabled family members. This corroborates the suggestion that the source of burnout resides in contextual factors and is independent of individual factors.^3^ Furthermore, a strong association between burnout and being a junior doctor was shown. This concurs with evidence that found burnout to be an early career phenomenon,^42^ with junior doctors experiencing an increased risk of burnout compared to their senior peers, because of inexperience in handling occupational stressors in an overburdened health system.^43,44,45,46,47^ It may also be likely that, in this study, burnout was not related to income band but was rather a function of the participants junior status. About a quarter of the participants (21%) used alcohol to manage WRS and feeling the need to use alcohol was associated with an increased risk of burnout. Since this study was cross-sectional in design, it is unclear whether alcohol use preceded or followed burnout. However, it is possible that participants were using alcohol, as a maladaptive coping mechanism, to ease the stressors associated with burnout.^19,48,49,50^

This study revealed significant associations between burnout scores and the job stressors of conflict at work, role ambiguity and role conflict, job satisfaction and support at work. Conflict at work, role ambiguity and role conflict have been found to be positively correlated with burnout.^39,45,51,52^ Some systematic reviews have described workload as a quantitative job demand, resulting in burnout.^12,14,19^ Surprisingly, in the current study, while participants reported high levels of workload, this was not significantly associated with burnout. This might be explained by the timing of the study, as participants had recovered from increasing demands and high workloads experienced during the earlier peak periods of the pandemic.^53^ In accordance with prior studies, we show that job satisfaction and support at work, by increasing job resources, assist in mitigating burnout in doctors.^14,18,19,53,54^ As expected, a negative correlation was shown between burnout and resilience score. While building resilience in doctors could assist in mitigating the job stressors, focussing only on resilience training of the individual doctor may distract from the broader organisational concerns that are determinants of burnout.^25,55,56,57^

Contrary to expectations, in this study, no statistically significant association was noted between burnout and COVID-19-related factors. Possible explanations for this may be that this study was conducted during the fifth wave of COVID-19, by which time the stress, uncertainty, lack of knowledge and preparedness, resulting from increased hospital admissions for COVID-19 had lessened.^8,58^ Vaccination was at this stage widely available, substantially mitigating risk of COVID-19 infection in the workplace. It may also be that the high burnout level in this study is less affected by COVID-19 and rather attributable to the ongoing organisational challenges faced by doctors working in these institutions.^30,31^ This study also explored the acceptability of preventative interventions at an organisational level to successfully reduce burnout among doctors. Poor working conditions and staff shortages have been identified in previous studies to be contributory to burnout, while management support was found to be protective.^12,18,19,40,42^ It is therefore not surprising that the interventions most supported by participants in this study include employing more staff, improving working conditions, improving equipment supply and management support. Interestingly, in contrast to the recommendations of global and local studies, provision of psychological counselling was one of the least supported interventions. This may be because of the Staff Psychologist providing debriefing sessions to doctors at one of the hospitals during the pandemic.

### Recommendations

To mitigate burnout, environmental controls recommended to enhance job satisfaction (supported by study participants) include improving staffing, working conditions, equipment supply, and intensifying management support.^16,19,42,52^ Administrative controls recommended to mitigate burnout involve developing clear policies to resolve role ambiguity and role conflict in doctors.^39,45,51^ Implementing guidelines to address organisational culture by promoting civility would ameliorate conflict at work and reduce discordancy among doctors and between doctors and other HCWs.^52,54^ Re-evaluation of human resource policies regarding overtime and the duration of shifts would reduce exhaustion experienced by doctors.^52^ To reduce burnout in the workplace, sustainable mental health interventions should be implemented as PPE.^59^ Therapeutic programmes addressing coping skills, resilience, mindfulness, debriefing, self-care strategies and mentoring should be offered to all doctors, particularly junior doctors and doctors with mental health conditions or substance use issues.^22,23,52,60^

To avoid the great variability in prevalence of previous burnout studies, it is advisable that future research be based on standardised, validated open-access instruments with universal cut-off scores, to afford a fair comparison of results. Investigating the relationship between burnout and job satisfaction would provide insight on the impact of burnout on turnover intentions of doctors, which would affect future healthcare needs in Gqeberha.

### Limitations

As a limitation, recall bias was unavoidable as data collected were based on participant’s self-reports. Response fatigue may be a reason for lack of participation, as another burnout survey was conducted a few months prior to this study. The Maslach Burnout Inventory (MBI), regarded as the gold standard in the diagnosis of burnout, is not free to use. There is no cost involved in the use of the OLBI. Therefore, because of financial limitations, the OLBI rather than the MBI was used in this study. Although the evidence for the validity of the OLBI, CD-RISC and NIOSH GJSQ in LMICs populations is limited, the inventories and scales have been widely used as validated tools. Because of the cross-sectional design, this study precludes the evaluation of temporality and causality of the observed associations since there was simultaneous collection of both exposures and outcome data. However, the findings of this study are similar to the results from local and international studies. It may therefore be suggested that the significant findings and associations in this study are a true reflection of factors associated with the development of burnout in this population.

## Conclusion

This study found the prevalence of burnout among doctors working in public hospitals in the Eastern Cape during the COVID-19 pandemic to be high, in comparison to studies done locally, and that occupational as well as organisational factors were primarily associated with increased risk for burnout. In an already low-resource setting, the burden of burnout among doctors constitutes a public health crisis with detrimental consequences for individual doctors, patients, healthcare facilities and society. Coronavirus disease 2019 has highlighted the existing systemic challenges faced by doctors working in public hospitals in South Africa. Consequently, to ensure the viability of the public healthcare system, policy makers and stakeholders will need to address the organisational factors rooted within healthcare systems which are contributing to burnout in doctors.
